# Untargeted Lipidomics of Erythrocytes under Simulated Microgravity Conditions

**DOI:** 10.3390/ijms24054379

**Published:** 2023-02-22

**Authors:** Cristina Manis, Antonio Murgia, Alessia Manca, Antonella Pantaleo, Giacomo Cao, Pierluigi Caboni

**Affiliations:** 1 Department of Life and Environmental Sciences, Cittadella Universitaria di Monserrato, Blocco A, Room 13, 09042 Monserrato, Italy; 2 Department of Mechanical, Chemical and Materials Engineering, University of Cagliari, Via Marengo 2, 09123 Cagliari, Italy; 3 Department of Biomedical Science, University of Sassari, Viale San Pietro, 07100 Sassari, Italy

**Keywords:** lipidomics, mass spectrometry, microgravity condition, erythrocytes

## Abstract

Lipidomics and metabolomics are nowadays widely used to provide promising insights into the pathophysiology of cellular stress disorders. Our study expands, with the use of a hyphenated ion mobility mass spectrometric platform, the understanding of the cellular processes and stress due to microgravity. By lipid profiling of human erythrocytes, we annotated complex lipids such as oxidized phosphocholines, phosphocholines bearing arachidonic in their moiety, as well as sphingomyelins and hexosyl ceramides associated with microgravity conditions. Overall, our findings give an insight into the molecular alterations and identify erythrocyte lipidomics signatures associated with microgravity conditions. If the present results are confirmed in future studies, they may help to develop suitable treatments for astronauts after return to Earth.

## 1. Introduction

The term microgravity in general refers to existing residual accelerations. When gravitation is the only force acting on an object, then it results is in free fall and hence it will experience microgravity [1]. Weightlessness is the state in which a body having a certain weight is balanced by another force or remains in free fall without feeling the effects of the atmosphere, equivalent to the situation faced by an astronaut aboard a spaceship. The effects of microgravity on human physiology have been studied extensively since the time of Yuri Gagarin (in 1961) who experienced the first man-on-board orbital flight, revealing profound implications for human health [2]. Despite the great interest and commitment of the scientific community, the mechanisms by which microgravity exerts its effects on the human body are not entirely clear.

Acute changes in normal physiology are typically seen in astronauts as a response and adaptation to abnormal environments. Such peculiar alterations require the attention of doctors and scientists [3]. In addition to alterations at the genetic level [4], microgravity experienced by space travellers also induces profound alterations at the cellular level. These alterations occurring at the cellular level are reflected in a series of pathological conditions such as a reduction of bone density, muscle atrophy, endocrine disorders, cognitive disorders and cardiovascular disfunctions, body fluid and electrolyte reduction, motion sickness, immune inhibition, and anaemia [5]. For these reasons, ground-based experiments simulating factors of spaceflight conditions are needed.

Microgravity is studied in several scientific and technological fields with the aim to highlight processes that on Earth are masked by the effects of the high gravitational field. Furthermore, the study of physiological processes in microgravity conditions allows the identification of the molecular mechanisms involved in different pathologies [6].

Roughly 350 people have experienced spaceflight in the past four decades, making it difficult to develop higher levels of clinical evidence to evaluate the effectiveness of space medicine interventions [7]. This great limitation, and the importance of studying the alterations at the cellular level that affect astronauts has led various research groups to study and build instruments capable of simulating space gravitational conditions on Earth.

The most employed methods to simulate microgravity are random positioning machines (RPM) and clinostats [8]. By controlled simultaneous rotating of the two axes, the clinostat cancels the cumulative gravity vector at the centre of the device, producing an environment with an average of 10^−3^ g. This is accomplished by the rotation of a chamber at the centre of the device to disperse the gravity vector uniformly within a spherical volume at a constant angular speed [9].

Bioactive lipid molecules known as signalling molecules, such as fatty acid, eicosanoids, diacylglycerol, phosphatidic acid, lysophosphatidic acid, ceramide, sphingosine, sphingosine-1-phosphate, phosphatidylinositol-3 phosphate, and cholesterol, are involved in the activation or regulation of different signalling pathways leading to apoptosis. Furthermore, alterations in the lipid composition determine membrane rigidity and fluidity, and play a crucial role in membrane organization, dynamics, and function [10]. Because of their biological role, lipids have been the subject of an intense area of research since the 1960s, which unfortunately was held back due to limited instrument platforms. Nowadays, lipidomics is considered an emerging science of fundamental importance for clarifying the biochemical pathways involved in several pathologies or cellular stress adaptations [11]. Advances in mass spectrometry (MS) and data processing, as well as the incorporation of soft ionization techniques, as ESI-MS2 method, has revolutionized the use of mass spectrometry, ushering this analytical tool in the field of lipidomics [12]. The lipidomics study can be applied as untargeted and targeted approaches, each with its own advantages and limitations [13]. Untargeted lipidomics focuses on the analysis of all detectable metabolites in a sample, including unknown chemicals, while targeted lipidomics is the measurement of defined groups of metabolites. While the strength of the targeted approach is validating one or more hypotheses, untargeted lipidomics allows for the discovery of new compounds that have led to a number of breakthroughs in understanding human disease risks [14]. Untargeted analyses can be performed with or without the addition of internal rules. When internal standards are added to samples, the method can provide pseudoconcentration results for particular metabolites or for metabolites with similar physicochemical properties (e.g., lipids). While these results are not truly quantitative, they may be accurate enough for case/control comparisons [15].

Despite the great relevance of the topic, currently few studies have been carried out to investigate the behaviour of lipids in erythrocyte samples cultured under simulated gravity conditions. In 2009, Ivanova et al. investigated blood samples from Russian cosmonauts by observing significant changes in the phospholipids class [16]. An increase in the percentage of phosphatidylcholine may be clearly associated with the increase in membrane rigidity. On the other hand, changes in the physicochemical properties of the plasma membrane of erythrocytes (microviscosity and permeability) can influence the efficiency of oxygen transfer, the state of the haemoglobin, and changes in the conformation of hematoporphyrin. Furthermore, changes in the in erythrocyte structure through an ultrastructural morphological analysis can be assessed by atomic force microscopy [17]. However, the study conducted by Ivanova’s team reported data deriving mainly from studies carried out after the end of a space flight, while only few data are related to changes that occur during a space flight. Moreover, lipid and phospholipid compositions of erythrocyte membranes were assayed by thin layer chromatography followed by densitometric measurement of stained dots. This technique provides information on the entire lipid class, but hardly allows the recognition of the specific lipid compounds. Since no data are reported on this subject, we decided to exploit the potential offered by chromatographic and mass spectrometry innovations to better understand the lipid modifications suffered by erythrocytes during simulated microgravity conditions. For this reason, with the aim to better understand which metabolic and/or structural changes occur in the erythrocytes subjected to low gravity, an experimental analysis of the erythrocytes’ lipid profile and their morphology under normal- and micro-g conditions was carried out following a recent investigation on the subject [18]. In detail, human erythrocytes were cultured in simulated gravity conditions, and they were collected at different times of clinorotation. For each sample, the organic phase was collected and analysed through ion mobility Q-TOF mass spectrometer (UHPLC-IM-QTOF-MS).

## 2. Results and Discussion

**Figure 1:**
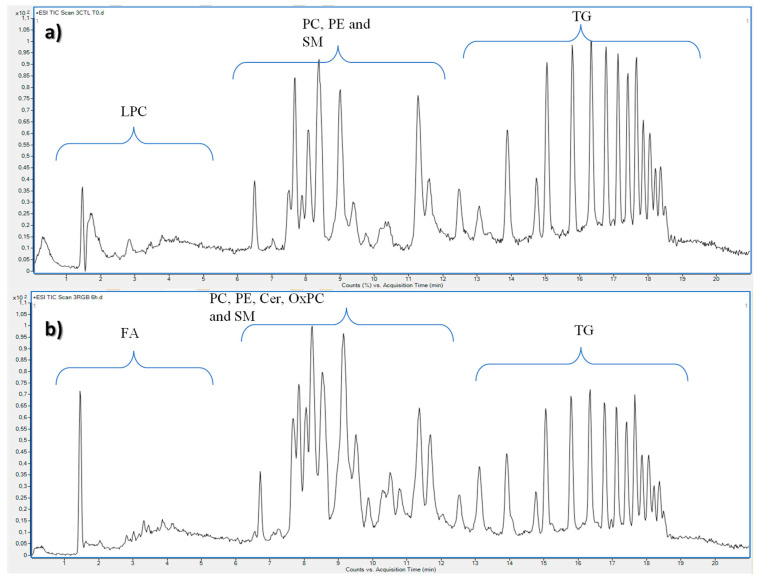
Representative total ion chromatograms of erythrocytes cultured in terrestrial gravity conditions and used as control (**a**) and compared with erythrocytes cultured in simulated gravity conditions (**b**) both collected after six hours of experiment.

To investigate the erythrocytes’ lipid profile after clinorotation and to describe possible variations among the different lipid categories, samples were analysed by IM-QTOF-LC/MS and representative total ion chromatograms are shown in Figure 1.

**Figure 2:**
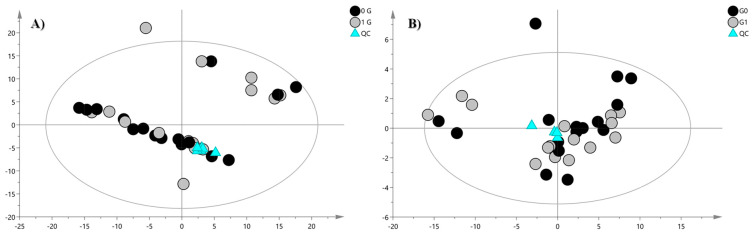
PCA score plot of (**A**) positive ion mode data and (**B**) negative ion mode data. The black circles represent the clinorotated samples, while grey circles represent control samples. The PCA analysis for PIA shows the following validation parameters: R2X = 0.808 and Q2 = 0.629; for the NIA model: R2X = 0.721, Q2 = 0.572.

Data processing yielded 215 and 160 features for the positive (PIA) and negative ionization analysis (NIA), respectively, which were subjected to multivariate statistical analysis (MVA). Chemical composition analysis indicated that the lipid fraction was composed of lipids from the following classes: free fatty acid (FA), lysophosphatidylcholines (LysoPC), phosphatidylcholines (PC), phosphatidylethanolamines (PE), sphingomyelins (SM), ceramides (Cer), and ether-linked oxidized phosphatidylcholine (EtherOxPC). Initially, to study sample distribution, to detect outliers, and to highlight differences or common features, a PCA was performed. The unsupervised analysis of both PIA and NIA features did not indicate any sample clustering correlated to clinorotation as shown in Figure 2.

**Figure 3:**
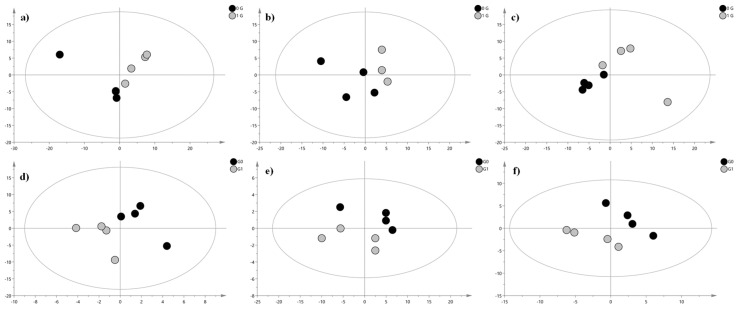
PLS-DA score plot. The grey circles represent the control samples, while black circles represent clinorotated erythrocytes samples. (**a**) 6 h clinorotated samples vs. 6 h control samples (PIA): R2X = 0.686, R2Y = 0.747, and Q2 = 0.434; (**b**) 9 h clinorotated samples vs. 9 h control samples (PIA): R2X = 0.849, R2Y = 0.945, and Q2 = 0.66; (**c**) 24 h clinorotated samples vs. 24 h control samples (PIA): R2X = 0.741, R2Y = 0.984, and Q2 = 0.421; (**d**) 6 h clinorotated samples vs. 6 h control samples (NIA): R2X = 0.776, R2Y = 0.994, and Q2 = 0.599; (**e**) 9 h clinorotated samples vs. 9 h control samples (NIA): R2X = 0.85, R2Y = 0.971, and Q2 = 0.147; (**f**) 24 h clinorotated samples vs. 24 h control samples (NIA): R2X = 0.692, R2Y = 0.995, and Q2 = 0.837.

However, the arrangement of the samples in the multivariate space appeared to be influenced by the time factor. Thus, to further limit the time factor influence, for each time point, we performed a PLS-DA. The validation parameters of the PIA and NIA models built for the samples collected at 6, 9, and 24 h are reported in the caption of the resulting plots (Figure 3).

**Figure 4:**
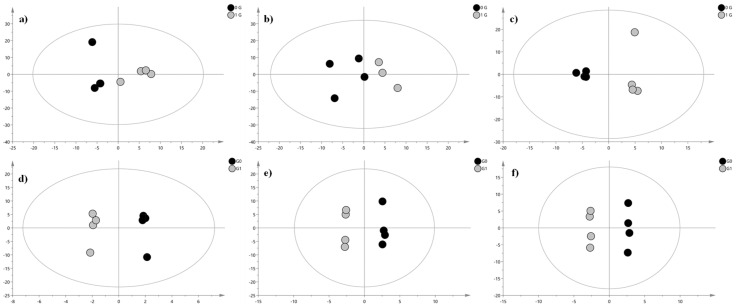
OPLS-DA score plot. The grey circles represent the control samples, while black circles represent clinorotated erythrocytes samples. (**a**) 6 h clinorotated samples vs. 6 h control samples (PIA): R2X = 0.868, R2Y= 0.862, and Q2 = 0.503; (**b**) 9 h clinorotated samples vs. 9 h control samples (PIA): R2X = 0.646, R2Y= 0.708, and Q2 = 0.199; (**c**) 24 h clinorotated samples vs. 24 h control samples (PIA): R2X = 0.741, R2Y= 0.984, and Q2 = 0.284; (**d**) 6 h clinorotated samples vs. 6 h control samples (NIA): R2X = 0.776, R2Y= 0.994, and Q2 = 0.482; (**e**) 9 h clinorotated samples vs. 9 h control samples (NIA): R2X = 0.883, R2Y= 0.998, and Q2 = 0.033; (**f**) 24 h clinorotated samples vs. 24 h control samples (NIA): R2X = 0.788, R2Y= 0.998, and Q2 = 0.721.

To identify metabolites that can discriminate for the two classes of samples (clinorotated vs. control samples), an OPLS-DA model of the IM-QTOF-LC/MS data was performed for each time point and for both polarities of acquisition. The OPLS-DA score plots are reported in Figure 4. In Table 1, we reported the discriminant metabolites between two classes and selected based on VIP value.

Using MS/MS fragmentation data and consulting the Metlin and Lipidomics libraries, we were able to tentatively identify the most discriminant metabolites as reported in Table 1.

Astronauts, after their return from space missions, manifest significant haematological alterations. Since the earliest space missions, symptoms such as structural alterations of red blood cells [18], anaemia [19], thrombocytopenia, [20,21], 10–17% reduction in plasma volume, and haemolysis [22] were reported. For these reasons, concern about the effects of space flight on haematological processes has been increasing. Several scientific studies allowed different theories to be proposed that may explain the alterations in the size and number of erythrocytes [23,24]. Recently, Trudel et al. showed in astronauts a degradation and 54% reduction in red blood cells [25]. Different factors can lead a human cell to programmed death, such as changes to lipid signal activity [26].

Human cells determine the characteristics of the plasma membrane bilayer by tightly controlling lipid composition and recruiting cytosolic proteins involved in structural functions or signal transduction [27]. The cell membrane is a lipid bilayer essentially formed by phospholipids, cholesterol, and glycolipids [28]. Small variations in percentage composition and molar ratio of the different classes of phospholipids and glycolipids might induce changes in the cell membrane’s fluidity and permeability. In particular, phospholipids are the main components of cell membranes and perform important biological functions.

From our results, it appears that after 6 h of clinorotation, levels of phosphocholines were increased in human erythrocytes. In particular, PC 18:1_20:4, PC 18:0_20:4, PC18:1_18:1, and PC 18:2_18:1 were found to be upregulated. Notably, PC with the arachidonic acid in their moiety were found discriminants. In particular, the proportion of sn-2-arachidonoyl-phosphatidylcholine (20:4-PC) has been shown to be inversely correlated with the activity of protein kinase B (Akt), an important kinase which promotes cell proliferation and survival. 20:4-PC reduces cell proliferation by interfering with the S-phase cell transition and by suppressing Akt downstream signalling and the expression of cyclin, such as LY294002, which is a specific inhibitor of the phosphatidylinositol-3-kinase/Akt [29]. At 9 and 24 h, erythrocytes showed other 20:4-PC upregulated: PC 18:2_20:4, PC 18:3_20:4 and PC 16:0_20:4, and PC 15:0_20:4, 15:1_20:4 and 16:0_20:4, respectively.

With the classical techniques of liquid chromatography coupled to mass spectrometry, the annotation of lipids and thus phosphocholine fatty acid composition with a good confidence interval is difficult due to the large variety of lipid species with different regiochemistry. In our study, the use of an analytical platform such as ion mobility coupled to mass spectrometry providing the collision cross section (CCS) value allows a better and more confident annotation of each metabolite. Each CCS was compared with an internal database and against the unified collision cross section compendium available on LipidMaps [30].

Additionally, a different fatty acid composition of membrane components can result in a greater sensitivity to peroxidative stress, with a consequent increase in membrane fragility. Phosphatidylcholine species containing polyunsaturated fatty acids in their moiety, particularly arachidonate, at the sn-2 position are susceptible to free radical oxidation [31]. An example is represented by 1-palmitoyl-2-arachidonoyl-sn-glycero-3-phosphatidylcholine (PC16.0_20:4), which is a common cell membrane constituent, and circulates within cholesterol particles. At 6 h of clinorotation, erythrocytes showed an upregulation of EtherOxPC 16:0_20:4, while the respective phosphocholine, PC 16:0_20:4, was found to be not discriminant. Simulated microgravity conditions increase reactive oxygen species (ROS) production in various cell types [32]. Generally, in microgravity conditions a different management of cellular resources was observed. In fact, in G0 conditions, there is a more rapid consumption of intracellular ATP, and an increase in ATP expulsion compared to cells cultured under terrestrial gravity conditions, coupled with a reducing power [8] resulting in a more oxidant environment.

Furthermore, inflammation and oxidative stress are associated with lipid peroxidation and the formation of bioactive lipids such as oxidized phosphocholines [33]. C-reactive protein (CRP), an acute-phase protein of hepatic origin that binds to specific structures expressed on the surface of dead or dying cells, promotes phagocytosis as macrophages may bind to these PC-oxidized species. Furthermore, recent studies demonstrate an enrichment of oxidized phosphatidylcholine in apoptotic cells [34]. Indeed, CRP can selectively bind on oxidized phosphatidylcholine but not on native phosphatidylcholine. In addition, oxidized phospholipids are recognized by macrophage scavenger, implying that these innate immune responses participate in cell clearance due to their proinflammatory properties [35]. Moreover, oxidized phosphatidylcholine, specifically oxidized-1-palmitoyl-2-arachidonoyl-sn-glycero-3-phosphatidylcholine (EtherOxPC 16:0_20:4), seems to be involved in ROS production. According to the study of Rouhanizadeh et al., EtherOxPC 16:0_20:4 was able to induce vascular endothelial superoxide production [36].

On the contrary, after 9 h of clinorotation, EtherOxPC(16:0_20:4) was downregulated, while PC 16:0_20:4 was upregulated, resulting non-discriminant after 24 h. These findings can lead us to hypothesize the complex adaptive response of cells.

Interestingly, several sphingomyelins were found to be downregulated for each experimental time point. This should not be surprising considering the mechanism of sphingomyelin synthesis. Indeed, sphingomyelinases (SMases) catalyse the hydrolysis of sphingomyelin to form ceramide and phosphocholine [37].

Taken together, these findings indicate that there are probably several mechanisms underlying spatial anaemia: inhibition of 20:4 PC-mediated cell proliferation and a simultaneous increase in pro-apoptotic signals.

## 3. Materials and Methods

### 3.1. Chemicals

Analytical LC-grade methanol, chloroform, acetonitrile, 2-propanol, and ammonium acetate and formiate were purchased from Sigma Aldrich (Milan, Italy). Bi-distilled water was obtained with a MilliQ purification system (Millipore, Milan, Italy). A SPLASH^®^ LIPIDOMIX^®^ standard component mixture was purchased from Sigma Aldrich (Milan, Italy): PC (15:0–18:1) (d7), PE (15:0–18:1) (d7), PS (15:0–18:1) (d7), PG (15:0–18:1) (d7), PI (15:0–18:1) (d7), PA (15:0–18:1) (d7), LPC (18:1) (d7), LPC 25, LPE (18:1) (d7), Chol Ester (18:1) (d7), MG (18:1) (d7), DG (15:0–18:1) (d7), TG ((15:0–18:1) (d7)-15:0)), SM (18:1) (d9), cholesterol (d7).

### 3.2. Cell Culture

Freshly drawn blood (Rh+) from 9 healthy adults of both sexes (men and women) was used, heparin was added and preserved in citrate-phosphate-dextrose with adenine (CPDA-1). Data are the average ± SD of three independent experiments. RBCs were separated from plasma and leukocytes by washing three times with phosphate-buffered saline (127 mM NaCl, 2.7 mM KCl, 8.1 mM Na_2_HPO_4_, 1.5 mM KH_2_PO_4_, 20 mM HEPES, 1 mM MgCl_2_, and pH 7.4) supplemented with 5 mM glucose (PBS glucose) to obtain packed cells. This study was conducted in accordance with Good Clinical Practice guidelines and the Declaration of Helsinki. No ethical approval has been requested as human blood samples were used only to sustain in vitro cultures and patients provided written, informed consent in ASL. 1-Sassari (Azienda Sanitaria Locale. 1-Sassari) centre before entering the study.

### 3.3. Microgravity Simulation

In order to study the effects caused by microgravity on human erythrocytes, the gravity simulator 3D Random Positioning Machine (RPM, Fokker Space, Netherlands) was used at the laboratory of the Department of Biomedical Sciences, University of Sassari, Sardinia, Italy. The 3D Random Positioning Machine (RPM) is a micro-weight (‘microgravity’) simulator based on the principle of ‘gravity-vector-averaging’, built by Dutch Space. The 3D RPM is constructed from two perpendicular frames that rotate independently. This setup was used to constantly change the mean value of the gravity vector to zero. In this way, the 3D RPM provides a simulated microgravity less than 10^−3^ g. The dimensions of the 3D RPM are limited to 1000 × 800 × 1000 mm (length × width × height). The 3D RPM is connected to a computer, and through a specific software the mode and speed of rotation were selected. Random Walk mode with an 80 degree/s (rpm) was chosen.

The red blood cell samples were carefully deposited in 2 mL tubes together with PBS-glucose (30% haematocrit, approximately 3.4 × 10^9^ cells) in a dedicated room at 37 °C. The control group samples were placed in the static bar at 1 g to undergo the same vibrations as the samples placed in µg conditions. Both control (1 g) and case (0 g) samples were collected after different time points (0, 6, 9, 24 h). Subsequently, the red blood cells were centrifuged and resuspended in 1 mL of lysis buffer [5 mM Na_2_HPO_4_, 1 mM EDTA (pH 8.0)] and stored at −20 °C until use for lipidomic analysis or fixed for confocal microscopic analysis.

### 3.4. Sample Preparation for UHPLC-IM-QTOF-MS Analysis

In order to investigate changes in the lipidome, analysis by UHPLC- IM-QTOF-MS requires the extraction of lipid content from cells [38]. An amount of 50 µL of human erythrocyte solution was extracted following the Folch procedure using 0.700 mL of a methanol and chloroform mixture (2/1, *v*/*v*). Samples were vortexed every 15 min up to 1 h, when 0.350 mL of chloroform and 0.150 mL of water were subsequently added. The solution thus obtained was centrifuged at 17,700 rcf for 10 min, and 0.600 mL of the organic layer was transferred into a glass vial and dried under a nitrogen stream. The dried chloroform phase was reconstituted with 50 μL of a methanol and chloroform mixture (1/1, *v*/*v*) and 75 μL isopropanol:acetonitrile:water mixture (2:1:1 *v*/*v*/*v*). Quality control (QC) samples were prepared taking an aliquot of 10 μL of each sample. All samples thus prepared were injected in UHPLC-IM-QTOF-MS/MS and acquired in negative ionization mode, while for positive ionization mode they were diluted in ratio 1:10.

### 3.5. UHPLC-IM-QTOF-MS/MS Analysis

The chloroform phase was analysed with a 6560-drift tube ion mobility LC-QTOF-MS coupled with an Agilent 1290 Infinity II LC system. An aliquot of 4.0 μL from each sample was injected in a Luna Omega C18, 1.6 μm, 100 mm × 2.1 mm chromatographic column (Phenomenex, Castel Maggiore (BO), Italy). The column was maintained at 50 °C at a flow rate of 0.4 mL/min. The mobile phase for positive ionization mode consisted of (A) 10 mM ammonium formate solution in 60% of milliQ water and 40% of acetonitrile and (B) 10 mM ammonium formate solution containing 90% of isopropanol and 10% of acetonitrile. In positive ionization mode, the chromatographic separation was obtained with the following gradient: initially, 80% of A, then a linear decrease from 80% to 50% of A in 2.1 min, then at 30% in 10 min. Subsequently, the mobile phase A was again decreased from 30% to 1% and stayed at this percentage for 1.9 min, and then was brought back to the initial conditions in 1 min. The mobile phase for the chromatographic separation in the negative ionization mode differed only for the use of 10 mM ammonium acetate instead of ammonium formate.

An Agilent jet stream technology source was operated in both positive and negative ion modes with the following parameters: gas temperature, 200 °C; gas flow (nitrogen) 10 L/min; nebulizer gas (nitrogen), 50 psig; sheath gas temperature, 300 °C; sheath gas flow, 12 L/min; capillary voltage 3500 V for positive and 3000 V for negative; nozzle voltage 0 V; fragmentor 150 V; skimmer 65 V, octapole RF 7550 V; mass range, 50−1700 *m*/*z*; capillary voltage, 3.5 kV; collision energy 20 eV in positive and 25 eV in negative mode, mass precursor per cycle = 3. High-purity nitrogen (99.999%) was used as a drift gas with a trap fill time and a trap release time of 2000 and 500 µs, respectively. Before the analysis, the instrument was calibrated using an Agilent tuning solution at the mass range of *m*/*z* 50–1700. Samples were evaporated with nitrogen at the pressure of 48 mTorr and at the temperature of 375 °C, while an Agilent reference mass mix for mass re-calibration was continuously injected during the run schedule.

The Agilent MassHunter LC/MS Acquisition console (revision B.09.00) from The MassHunter suite was used for data acquisition.

### 3.6. Data Analysis

Data acquired with the Agilent 6560 DTIM Q-TOF LC-MS were pre-processed with the software MassHunter Workstation suite (Agilent Technologies, Santa Clara, CA, USA). This software (Mass Profiler 10.0) allowed us to perform mass re-calibration, DTCCSN2 re-calibration, time alignment, and deconvolution of signals, yielding a matrix containing all features present across all samples. The removal of background noise and unrelated ions was performed by a recursive feature extraction tool, yielding a matrix containing all the features present across all samples. Furthermore, to eliminate non-specific information, data matrix quality assurance was performed. This filtered matrix was then subjected to multivariate statistical analysis using SIMCA software 15.0 (Umetrics, Umeå, Sweden).

First, a principal component analysis (PCA) was carried out. This unsupervised analysis allows an observation of samples and variables distribution in the multivariate space on the basis of their similarity and dissimilarity. This was followed by partial least square-discriminant analysis (PLS-DA) with its orthogonal extension (OPLS-DA), which was used as a classificatory model to visualize and evaluate the differences between sample classes.

## 4. Conclusions

Spatial anaemia in astronauts has been noted since the earliest space missions, while the contributing mechanisms during space flight remained unclear. To investigate the molecular mechanisms that induce a reduction in the number of erythrocytes during spaceflight, we decided to analyse the lipid profile of human erythrocytes under microgravity conditions. Thanks to the advancement of hyphenated techniques and mass analysers, we were able to identify biologically active complex lipids susceptible to microgravity, allowing new possible hypotheses that explain the anaemia experienced by astronauts.

In more detail, lipidomic analysis of erythrocytes revealed a double mechanism that generates the reduction in the number of red blood cells. On one hand, there is an increase in the levels of 20:4 PC, reducing cellular proliferation. On the other hand, the increase in the levels of EtherOxPC 16:0_20:4 stimulates the immune response by attracting the C-reactive protein and macrophages and induces an increase in ROS production [33,34]. ROS increase caused by microgravity inevitably induces mitochondrial damage and dysfunction as indicated by the accumulation of HexCer lipid species in clinorotated erythrocytes. This accumulation acts as a pro-apoptotic signal condemning the erythrocytes to death.

In this study, we reported a set of lipid discriminants or potential biomarkers linked to microgravity exposure with the aim to explore in the future specific lipid pathways and form the foundation in the development of novel therapeutics in hopes of reducing the effects of space flights. However, further studies are needed to accurately measure lipids in erythrocytes samples to better understand the clinical effects of microgravity.

## Figures and Tables

**Table 1:** Discriminant metabolites of human erythrocyte samples annotated by LC/DTIM-QTOF-MS.

Lipid	Adduct	*m/z Experimental*	*m/z Theoretical*	Δ (ppm)	RT (min)	Fatty Acid Composition	^DT^CCS_N2_ (Å^2^)	VIP	Significance Level	Regulation in G0 Cells
**6 h**										
LysoPC16:1	+H^+^	494.3216	494.3241	5.1	6.55	16:1	231.58	1.73	ns	up
PC 32:2	+H^+^	730.5394	730.5381	1.3	7.77	32:2	286.75	1.71	**	up
PC 38:5	+H^+^	808.5871	808.5851	2.0	8.05	18:1, 20:4	292.87	1.50	**	up
PC 33:5	+H^+^	738.5070	738.5068	0.3	7.74		287.33	1.26	ns	down
SM d34:0	+H^+^	705.5930	705.5905	3.5	8.01	16:0, 18:0	285.87	1.10	*	down
PC 38:4	+H^+^	810.6021	810.6007	1.7	9.23	18:0, 20:4	295.11	1.05	**	up
PC 36:2	+H^+^	786.6031	786.6007	3.0	9.43	18:1, 18:1	291.56	1.00	*	up
SM d 38:1	+H^+^	759.6328	759.6375	6.0	11.31		297.55	1.00	**	down
PC 36:1	+H^+^	788.6192	788.6164	3.2	10.43		293.88	0.99	*	up
FA 16:2	-H^−^	251.2021	251.2017	1.6	3.59	16:2	114.95	1.38	**	down
HexCer_AP t37:1	+(C_2_H_3_O_2_)^−^	832.6152	832.6164	1.5	10.43	22:1, 15:0	296.27	1.46	**	up
Etn-1-P-Cer 32:1	-H^−^	687.5469	687.5446	3.4	7.51	14:1, 18:0	265.47	1.13	***	up
EtherOxPC 36:4e + 1O	+(CHO_2_)^−^	828.5761	828.5781	2.4	8.3	16:0, 20:4	293.50	1.07	*	up
SM d40:1	+(CHO_2_)^−^	831.6632	831.6597	4	11.66		301.49	1.06	ns	up
PE 36:3	-H^−^	740.5249	740.5236	2	7.50	18:2, 18:1	270.58	1.02	ns	up
**9 h**										
PC 38:6	+H^+^	806.5700	806.5694	0.74	8.48	18:2, 20:4	293.14	1.79	*	up
SM d 38:1	+H^+^	759.6328	759.6375	6.0	11.31		297.55	1.75	**	down
PC 35:5	+H^+^	766.5399	766.5381	2.3	8.33	15:1, 20:4	280.17	1.73	***	up
PE 36:1	+H^+^	746.5712	746.5694	2.1	10.71	18:0, 18:1	281.87	1.71	**	up
PC 36:5	+H^+^	780.5541	780.5538	0.3	8.11	16:1, 20:4	228.33	1.70	**	up
PC 32:0	+H^+^	734.572	734.5694	3.5	8.92	16:0, 16:0	284.83	1.64	**	up
PC 36:2	+H^+^	786.6031	786.6007	3.0	9.25	18:1, 18:1	291.56	1.63	**	up
SM d34:0	+H^+^	705.5930	705.5905	3.5	8.01	16:0, 18:0	285.87	1.61	**	up
PC 35:5	+H^+^	766.5407	766.5381	3.4	8.51		288.55	1.60	**	down
PE 38:6	+H^+^	764.524	764.5225	2.0	7.89	16:0, 22:6	279.42	1.59	**	up
PC 36:3	+H^+^	784.5872	784.5851	2.7	8.25	18:1, 18:2	289.42	1.57	**	up
PC 35:4	+H^+^	768.5593	768.5538	7.0	8.73	15:0, 20:4	289.52	1.56	**	up
SM d44:5	+H^+^	835.6691	835.6688	1.0	11.61	14:3, 30:2	305.48	1.52	**	up
SM 42:3	+H^+^	811.6713	811.6688	3.1	10.49		302.65	1.48	**	up
PC 38:7	+H^+^	804.554	804.5 538	0.3	7.93	18:3, 20:4	292.2	1.45	ns	up
PC 34:0	+H^+^	762.6032	762.6007	3.3	10.27		290.86	1.38	ns	up
PC 36:5	+H^+^	780.5541	780.5538	0.3	8.11		289.42	1.37	*	up
PC 36:4	+H^+^	782.5701	782.5694	1.0	9.08	16:0, 20:4	229.67	1.36	**	up
PC 34:3	+H^+^	756.554	759.5538	0.3	8.92	18:1, 16:2	286.35	1.33	*	up
PE 34:2	+H^+^	716.5251	716.5225	3.5	8.37	16:0, 18:2	273.06	1.23	ns	up
PC 36:1	+H^+^	788.6192	788.6164	3.2	10.43		293.88	1.21	*	up
SM d42:2	+H^+^	813.6870	813.6844	3.2	11.61		305.35	1.21	**	up
PC 38:5	+H^+^	808.5870	808.5851	2.3	8.04	16:0, 22:5	292.87	1.20	ns	up
PC 33:5	+H^+^	738.5070	738.5068	0.3	7.74		287.33	1.07	*	down
PE 36:3	+H^+^	742.5399	742.5381	2.5	8.51	16:0, 20:3	276.61	1.04	ns	up
PE 36:4	+H^+^	740.5239	740.5225	2.0	8.19	16:0, 20:4	276.93	1.03	**	up
SM d42:2	+H^+^	813.6870	813.6844	3.2	11.61		305.03	1.00	**	up
EtherOxPC 36:4e + 1O	+(CHO_2_)^−^	828.5761	828.5781	2.4	8.3	16:0, 20:4	293.50	1.56	*	down
PE 36:2	-H^−^	742.5403	742.5392	1.5	9.55	18:1, 18:1	271.52	1.16	**	up
PC 38:6	+(CHO_2_)^−^	850.5613	850.5604	1.1	7.62	18:2, 20:4	296.34	1.01	ns	up
**24 h**										
PC 35:4	+H^+^	768.5593	768.5538	7.0	8.73	15:0, 20:4	289.52	1.84	**	up
PE 36:4	+H^+^	740.5239	740.5225	2.0	8.19	16:0, 20:4	276.93	1.84	**	up
PC 36:2	+H^+^	786.6031	786.6007	3.0	9.25	18:1, 18:1	291.56	1.64	**	up
PC 32:0	+H^+^	734.572	734.5694	3.5	8.92	16:0, 16:0	284.83	1.61	ns	up
SM d42:2	+H^+^	813.6870	813.6844	3.2	11.98		305.03	1.46	*	up
SM 42:3	+H^+^	811.6713	811.6688	3.1	10.49		302.65	1.40	**	up
PC 36:3	+H^+^	784.5872	784.5851	2.7	8.25	18:1, 18:2	289.42	1.35	*	up
PE 36:1	+H^+^	746.5712	746.5694	2.1	10.71	18:0, 18:1	281.87	1.34	ns	up
PE 38:6	+H^+^	764.524	764.5225	2.0	7.89	18:2, 20:4	279.42	1.21	**	up
PC 36:4	+H^+^	782.5701	782.5694	1.0	9.08	16:0, 20:4	229.67	1.20	***	up
PC 36:5	+H^+^	780.5541	780.5538	0.3	8.11		289.42	1.20	*	up
PC 38:4	+H^+^	810.6021	810.6007	1.7	9.23	18:0, 20:4	295.11	1.19	**	up
PC 34:0	+H^+^	762.6032	762.6007	3.3	10.27		290.86	1.13	*	up
PC 35:5	+H^+^	766.5399	766.5381	2.3	8.33	15:1, 20:4	280.17	1.11	*	down
PE 36:3	+H^+^	742.5399	742.5381	2.5	8.52	16:0, 20:3	276.61	1.11	ns	up
SM d44:5	+H^+^	835.6691	835.6688	1.0	11.61	14:3, 30:2	305.48	1.09	**	up
SM d42:2	+H^+^	813.6870	813.6844	3.2	11.62		305.35	1.05	ns	up
PC 35:5	+H^+^	766.5407	766.5381	3.4	8.51		288.55	1.03	*	down
LysoPC16:1	+H^+^	494.3216	494.3241	5.1	6.55	16:1	231.58	1.01	*	up
FA 16:2	-H^−^	251.2021	251.2017	1.6	3.59	16:2	114.95	1.99	ns	up
Cer 42:1	+(CHO_2_)^−^	694.6369	694.6355	2.1	15.27	18:1, 24:0	278.31	1.02	*	down
HexCer_AP t37:2	+(C_2_H_3_O_2_)^−^	830.5979	830.5999	2.5	9.42	22:1, 15:1	295.07	1.01	ns	down
HexCer_AP t37:1	+(C_2_H_3_O_2_)^−^	832.6152	832.6164	1.5	10.43	22:1, 15:0	296.27	1.01	ns	down

ns: not significant; *** *p* value < 0.001; ** *p* value < 0.01; * *p* value < 0.05.

## Data Availability

On request.

## Abbreviations

fatty acid (FA); phosphatidylethanolamine (PE); ether-linked phosphatidylcholine (Ether-PC); phosphatidylcholine (PC); lysophosphocholine (LPC); sphingomyelin (SM).
